# Evaluating the Urinary Exosome microRNA Profile of von Hippel Lindau Syndrome Patients with Clear Cell Renal Cell Carcinoma

**DOI:** 10.3390/genes15070905

**Published:** 2024-07-11

**Authors:** Beatriz Walter-Rodriguez, Christopher J. Ricketts, W. Marston Linehan, Maria J. Merino

**Affiliations:** 1 Laboratory of Pathology, Center for Cancer Research, National Cancer Institute, National Institutes of Health, Bethesda, MD 20892, USA; beatriz.walterrodriquez@nih.gov; 2 Urologic Oncology Branch, Center for Cancer Research, National Cancer Institute, National Institutes of Health, Bethesda, MD 20892, USA; chris.ricketts@nih.gov (C.J.R.); wml@nih.gov (W.M.L.)

**Keywords:** urinary exosomes, miRNA, VHL, ccRCC, renal cell carcinoma, von Hippel Lindau, liquid biopsy, molecular markers

## Abstract

Introduction: Renal cell carcinoma is one of the ten more common malignant tumors worldwide, with a high incidence and mortality rate. Kidney cancer frequently presents at an advanced stage, and it is almost invariably fatal. Much progress has been made in identifying molecular targets for therapy in the hope of improving survival rates, but still, we have no good markers for early detection or progression of the disease. Von Hippel Lindau syndrome (VHL) is an autosomal dominant cancer hereditary syndrome in which affected individuals are at risk of developing bilateral and multifocal renal cell carcinomas (RCC) as well as other tumors. These patients provide an ideal platform to investigate the potential of urinary exosomal miRNA biomarkers in the early development of ccRCC, as these patients are regularly imaged and tumors are actively monitored until the tumor reaches 3 cm before surgical excision. This allows for pre- and post-surgical urine collection and comparison to excised tumor tissues. Studying different biomarkers in urine can provide comprehensive molecular profiling available to patients and physicians and can be a great source of additional tumor genetic information. Methods: Pre- and postoperative urine samples were obtained from a cohort of VHL patients undergoing surveillance and surgical excision of ccRCCs, and exosomes were extracted. MicroRNA-Seq analysis was performed on miRNA extracted from both urine-derived exosomes and FFPE material from excised ccRCCs. Results: MicroRNA-Seq analysis highlighted a significant difference in the urinary exosome-derived miRNA expression profiles between VHL patients and normal control individuals. This included decreased expression of the miR-320 family, such as miR-320a, known to be decreased in sporadic ccRCC and suppressed by the HIF1α transcription factor activated by the loss of the VHL gene. MiR-542-5p represented a potential marker of VHL-associated ccRCC that was lowly expressed in normal control urinary exosomes, significantly increased in the preoperative urinary exosomes of tumor-bearing VHL patients, and subsequently reduced to normal levels of expression after tumor excision. In concordance with this, the expression of miR-542-5p was increased in the VHL-associated ccRCC in comparison to the normal kidney. Conclusions: This study shows the potential for miRNA profiling of exosomes from readily available biofluids to both distinguish VHL patient urine from normal control urine microRNAs and to provide biomarkers for the presence of VHL syndrome-associated ccRCC. Further validation studies are necessary to demonstrate the utility of urinary exosome-derived miRNAs as biomarkers in kidney cancer.

## 1. Introduction

Kidney cancer is among the 10 most common cancers in both men and women, representing approximately 4.1% of all adult malignant tumors, with an estimated 81,610 new cases and 14,390 deaths in 2024 and a 5-year relative survival of 78.1% (https://seer.cancer.gov/statfacts/html/kidrp.html, accessed on 9 July 2024). The majority of kidney cancer occurs sporadically, but numerous hereditary cancer susceptibility syndromes account for approximately 5% of cases and are associated with known germline genetic alterations. Understanding these rarer syndromic kidney cancers has provided invaluable insights into the biology and genetics of kidney cancer in general [1,2].

The most common form of hereditary kidney cancer susceptibility is von Hippel Lindau disease (VHL), which follows a pattern of autosomal dominant inheritance caused by heterozygous germline inactivation of the *VHL* gene encoded on the short arm of chromosome 3 (3p25.3) [1,3]. VHL patients are clinically characterized by an increased risk of developing multiple benign and malignant tumors within various organs, including retinal and central nervous system hemangioblastomas, pheochromocytomas, or paragangliomas, and clear cell renal cell carcinomas (ccRCC) [4,5,6]. Approximately 70% of VHL patients will develop ccRCC over their lifetimes, and patients are regularly imaged to provide early diagnosis. VHL patients with kidney lesions are actively monitored until their largest tumor reaches 3 cm and surgery is performed, and then the patients remain on active management with continual imaging.

Liquid biopsy is a non-invasive technique that can utilize different biofluids, including saliva and urine, as a source for the detection of circulating tumor cells or tumor cell-derived genetic materials, such as exosomes, to aid in diagnosis. Extracellular vehicles (EVs), including exosomes, can shuttle molecular material from tumor cells around the body and carry genetic material such as mRNAs and microRNAs (miRNA) that can be distinct to these donor tumor cells [7,8]. Extracting exosome-derived miRNAs by liquid biopsy from cancer patients is an attractive prospect due to miRNA being highly stable and relatively easy to quantify in circulating form [8,9,10].

Dysregulation of miRNA expression is a major aspect of the altered biology of cancer cells that can occur due to various mechanisms, including chromosomal copy number alteration and epigenetic changes [11,12,13]. MiRNAs can be oncogenic or act as tumor suppressors, such as miR-16 and miR-15, that are encoded at chromosome 13q14.3, a region commonly deleted in various cancer types, including chronic lymphocytic leukemia (CLL) [12,13,14]. Understanding both the miRNA profile of a tumor and how this is reflected in the liquid biopsy miRNA profile is important and likely specific to each tumor type. In sporadic RCC, initial miRNA analysis of both serum and urine highlighted several differentially expressed miRNAs between sporadic RCC patients and healthy controls, including miR-15a, miR-378, miR-451, and miR-1223 [15,16,17]. More recently, this was refined in multiple studies to the evaluation of urinary exosomal miRNAs where differential expression of miR-126-3p (in combination with either miR-449a or miR-34b-5p), miR-30c-5p, miR-210 and miR-1233, or miR-92a-1-5p, miR-149-3p, and miR-424-3p could distinguish between ccRCC patients and normal controls [18,19,20,21]. In cancer, miRNAs can be considered both potential biomarkers for the presence or progression of disease and potential therapeutic targets. It is possible to utilize both synthetic miRNA mimics to counteract down-regulation of tumor-suppressing miRNA, such as the miR-16 mimic TargomiR, and to target overexpressed miRNA with antisense-based oligos [12,22]. Therefore, improved understanding of miRNA profiles within specific tumor types can be beneficial for multiple reasons.

While sporadic forms of ccRCC have been previously investigated, the urinary exosomal miRNA profile for ccRCC associated with VHL syndrome has yet to be assessed. VHL patients provide an ideal platform to investigate urinary exosomal markers of early development of ccRCC as the patients are regularly imaged and any resultant tumors are actively surveilled until the largest tumor reaches 3 cm and surgery is performed. This allows for preoperative urine samples to be taken, excised tumors to be evaluated, and postoperative urine samples to be evaluated as the patient continues to be managed. The aim of this study was to investigate the urinary exosomal miRNA expression profile within VHL patients and identify any potential noninvasive biomarkers for VHL-associated ccRCC by comparison between preoperative and postoperative urine samples and evaluation of excised VHL-associated ccRCC tumors.

## 2. Materials and Methods

### 2.1. Patients’ Enrollment and Sample Collection

A total of 34 patients with VHL-associated ccRCC were included in our study. Patient recruitment, subsequent tissue procurement and use, and publication of data were approved by the Institutional Review Board of the National Cancer Institute on the NCI-97-C-0147 or NCI-89-C-0086 protocols. All patients provided written, informed consent for both protocols. In total, 67 samples were analyzed, including 43 urine specimens, 13 FFPE kidney tumor specimens closest to the urine collection time (September 2021 to January 2023), 10 urine samples from healthy donors, and one pool of normal kidney tissue (FFPE). Urine samples were collected at an outpatient clinic.

### 2.2. Urinary Exosome Isolation

Fresh, mid-stream urine samples were obtained at the outpatient clinic after collection instructions were given to the subjects. Samples were kept at 4 °C and processed within 4–6 h after the collection time (see Supplementary Table S1 for urine collection sheets with the information regarding collection, processing, and storage conditions as recommended for urinary EV research by the International Society for Extracellular Vesicles (ISEV) in 2021 [23]). A total volume of the urine was aliquoted into 15 mL conic polypropylene tubes and centrifuged at 3000× *g* 15 min in an Allegra X-30 centrifuge (Beckman Coulter Inc., Sykesville, MD, USA) to obtain the cell-free urine (CFU). CFU was then separated from the cell pellets, transferred into a new conic tube, and frozen at −80 °C until use. From the CFU, exosomes were isolated based on size-exclusion chromatography (SEC) using the Exo-Urine™ EV Isolation Kit (Cat. No. EXOU100A-1, System Biosciences, SBI, Palo Alto, CA, USA) per the manufacturer’s protocol. The exosome eluate, which contained the desired EV fractions, was then processed for RNA isolation.

### 2.3. Urinary Exosome Characterization

The characterization of the exosomes was performed using antibody-based array assays and Western blot techniques. For the antibody-based array assay, a total of 8 samples (5 pooled urine exosomes from 5 VHL patients and 3 pooled normal urine exosomes) were evaluated using the Exo-check^®^ antibody array (Cat. No. EXORAY210B-8, System Biosciences, SBI, Palo Alto, CA, USA) to confirm the presence of exosomes from the final EV fraction obtained from the isolation. Each array has 12 pre-printed spots, 8 spots with antibodies for known exosome markers (CD81, CD63, ALIX, FLOT-1 ICAM, EpCAM, ANXA5, and TSG101), 2 HRP positive control spots, and 1 blank negative control spot. GM130, an antibody that predicts cellular contamination from organelles, is also included. After membrane incubation with 50 ng of total protein, a signal was detected by a secondary-HRP-conjugated antibody, and the image was obtained in a Western blot chemiluminescent imaging system (Bio-Rad Laboratories, Inc., Philadelphia, PA, USA). Semi-quantitative and qualitative analysis indicated the presence of exosome protein markers when the spots labeled with the proteins were detected (Supplementary Table S2 includes results with the list of proteins labeled in the array).

Western blot analysis of the EV fraction was also performed to confirm the presence of exosomes. A total of 0.2 mg/mL of exosome lysate from each of the available samples and from the control urine exosome were reconstituted in 1X RIPA lysis buffer for further analysis. Equal amounts of protein from each sample were prepared, denatured, and combined with Luminol-S and Peroxide before being loaded into the microplate (separation module of 12–230 KDa) along with the antibody exosome panel of CD63 [EPR5702] (ab134045), CD81 [EPR4244] (ab109201), CD9 [EPR23105-125] (ab263019), and TSG101 [EPR7130(B)] (ab125011) (Abcam Inc., Cambridge, MA, USA), and the wash buffer. The capillary cartridge was run into the automated Western Immunoassay system (Jess Protein Simple, Biotechne, Minneapolis, MN, USA), and the target proteins were identified using an HRP-conjugated secondary antibody with the chemiluminescent substrate. The signal was detected and quantified (Supplementary Figure S1B shows a representative image of the Western blot).

### 2.4. Urinary Exosome RNA Extraction and Quality Control

The EV fraction was processed to extract total RNA using the SeraMir^®^ Exosome RNA Purification Column Kit following the protocol recommendations (Cat. No. RA806A-1, System Biosciences, SBI, Palo Alto, CA, USA). For quality control, gDNA genomic contamination and RNA quantification for each sample were measured in a Bioanalyzer 2000 RNA 6000 Pico assay (Agilent Technologies, Santa Clara, CA, USA). Samples with more than 1 ng/5 µL were selected for library preparation for Next Generation Analysis (NGS).

### 2.5. RNA Extraction from FFPE ccRCCs

Available FFPE ccRCC tumor samples (*n* = 13) from the same cohort of 34 patients were retrieved, and 5 micron-thick samples mounted on glass slides were needle-manually microdissected by an experienced pathologist. The ccRCC cells were collected, and the total RNA was extracted according to product recommendations using the RNeasy FFPE Kit (Cat. No. 73504, QIAGEN, Germantown, MD, USA). One pool of FFPE normal kidney from non-renal tumor tissue was also microdissected and included. The quality control and quantification were measured in the bioanalyzer before all the samples proceeded for NGS.

### 2.6. Library Preparation and Sequencing

Library preparation (QIAseq^®^ miRNA Library—Cat. No. 331502, QIAGEN, Germantown, MD, USA) for microRNA next-generation sequencing (NGS) analysis was carried out by the Frederick National Laboratory for Cancer Research Sequencing Facility (CCR, NIH) on the Ilumina platform (Illumina, Inc., San Diego, CA, USA). MicroRNA-Seq samples were pooled and sequenced on NovaSeq 6000 S1 using QIAseq miRNA Library Prep and single-end sequencing.

### 2.7. Statistical Analysis

The FASTQ data from Illumina^®^ were used for quality checking with Multiqc v1.13 (Illumina, Inc., San Diego, CA, USA). Samples were trimmed using Trimmomatic before alignment, followed by miRDeep2 analysis with the hg38 reference. Bioinformatic and statistical analyses for NGS raw data were performed on the workbook bulk-sequencing (RNA-seq) on the NIH Integrated Data Analysis Platform (NIDAP) for filtering and QC, PCA, expression heatmaps, differential expression analysis, and volcano plots. Differential expression analysis was predicated on the criteria of a *p*-value < 0.001 and |log_2_FC| > 1. Additional statistical analysis was performed using GraphPad Prism 9.

## 3. Results

### 3.1. Clinicopathological Features of Study Samples

A total of 34 patients with VHL disease and evidence of RCC at our starting time point were included in our study. Preoperative urine samples were acquired from patients during their surveillance, and postoperative urine samples were acquired after surgery whenever feasible. Patients had a median age of 44 years (range 21–72 years) and were more frequently male (20/34, 58.8%). In all the cases, kidney tumors had been diagnosed by imaging, and at least one surgery to remove the tumor(s) was performed during the time period in which urine samples were collected. These excised kidney tumor samples were evaluated by an experienced pathologist, and the ccRCC diagnosis was confirmed in all the cases. Moreover, 34 kidney tumors were graded according to the World Health Organization/International Society of Urological Pathology (WHO/ISUP), with 25 tumors designated as grade 2, 8 tumors designated as grade 3, and a single tumor designated as grade 4. Three cases demonstrated distant metastasis. A summary of clinicopathological findings is in Table 1. In these patients, exosomes were isolated from 15 preoperative urine samples and 28 postoperative urine samples. Due to the clinical phenotype of VHL syndrome, in which many ccRCC tumors develop in both kidneys over time, it is possible that the postoperative patients still retain some tumor burden relating to tumors that were too small to image and/or remove. Moreover, the postoperative urine samples do not necessarily represent a complete absence of disease but should represent a significant reduction in detectable tumor burden. In addition, exosomes were isolated from 10 normal donor individuals to provide a control group.

### 3.2. Characterization of Exosome Isolates from Urine

A subset of the exosome isolation, representing five VHL urine samples and three normal donor urine samples, was evaluated by Exo-Check Exosome Antibody Arrays. The intensity of signal detected in each antibody array spot was scored from negative to 3+ according to visual signal intensity, with both positive and negative controls reacting as expected (Supplementary Figure S1A and Table S2). CD81 was weakly positive (+), whereas CD63 was strongly and consistently positive (+++) in all eight samples from both VHL and normal urine samples. GM130 was either weakly or moderately positive in both VHL (2/5 ++, 2/5 +) and normal (2/3 +) urine samples; two spots were not informative due to the diffusion of signals from adjacent markers. FLOT-1 showed weak or no positivity in both VHL (3/5 +, 2/5 −) and normal (2/3 +, 1/3 −) urine samples, as did EpCAM (VHL: −2/5 +, 3/5 −, normal: 1/3 +, 2/3 −). ICAM showed strong positivity in both VHL (2/5 +++, 3/5 ++) and normal (2/3 +++, 1/3 −) urine samples, as did ANXA5 (VHL: −4/5 +++, 1/5 ++, normal: 2/3 +++, 1/3 −) and TSG101 (VHL: −3/5 +++, 2/5 ++, normal: 2/3 +++, 1/3 −). ALIX was either weakly or moderately positive in both VHL (1/5 ++, 4/5 +) and normal (1/5 ++, 4/5 +) urine samples. These results of the array indicate the presence of exosomes in our samples by consistent and positive detection of the Tetraspanin family exosomal markers CD81 and CD63 (Supplementary Figure S1A and Table S2). Further exosome presence validation was conducted by Western blot analyses of CD9, CD63, CD81, and TSG101 on selected example samples (Supplementary Figure S1B). The expression of TSG101 in the exosome preparations indicates some contamination of material derived from other cell compartments.

### 3.3. Comparison of Urinary Exosome microRNA Profiles between VHL Patients and Normal Controls

**Figure 1:**
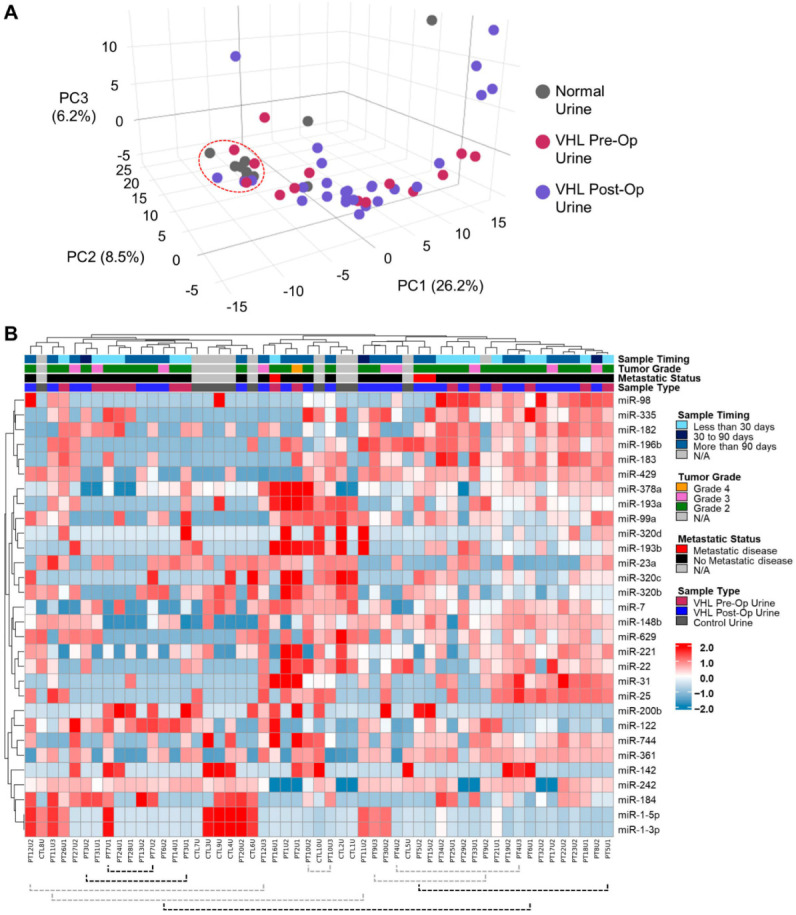
Differential miRNA expression in VHL-associated ccRCCs. (**A**) A 3D PCA analysis of the urinary exosome-derived miRNAs from 10 normal controls and 43 VHL patient samples, including 15 preoperative and 28 postoperative urine samples. A cluster of the majority of normal controls is indicated by a dashed red circle. (**B**) Heatmap representation and dendrogram for the 30 most variable miRNAs across the cohort of samples. The type and timing of procurement for each sample, along with the patient’s tumor grade and metastatic status, are shown. Paired samples representing both pre- and postoperative samples are highlighted with black dashed brackets, and pairs representing multiple postoperative samples are highlighted with gray dashed brackets.

The exosome-derived miRNA profiles were acquired from the urine samples of 34 VHL patients, consisting of 15 preoperative urine samples, 28 postoperative urine samples, and 10 normal control donors. PCA analysis demonstrated concordance in the miRNA profile for most of the control donor exosomes, with a few outliers (Figure 1A). The majority of VHL urine samples were clustered away from the normal controls, demonstrating a distinct signal was present in the VHL patient urine, but no obvious clustering differentiated the preoperative and postoperative samples (Figure 1A). Heatmap analysis of the 30 most variable miRNAs confirmed the distinction between the control urine samples and the VHL urine samples but also highlighted a great degree of variation between samples (Figure 1B). Nine patients had two samples analyzed, and distinct differences were observed between both pairs involving pre- and postoperative urine samples and those representing multiple postoperative urine samples from the same patient (Figure 1B). The nine samples derived from patients with grade 3 or higher tumors showed no distinct clustering, and the three patients with metastasis were too few to evaluate (Figure 1B).

**Figure 2:**
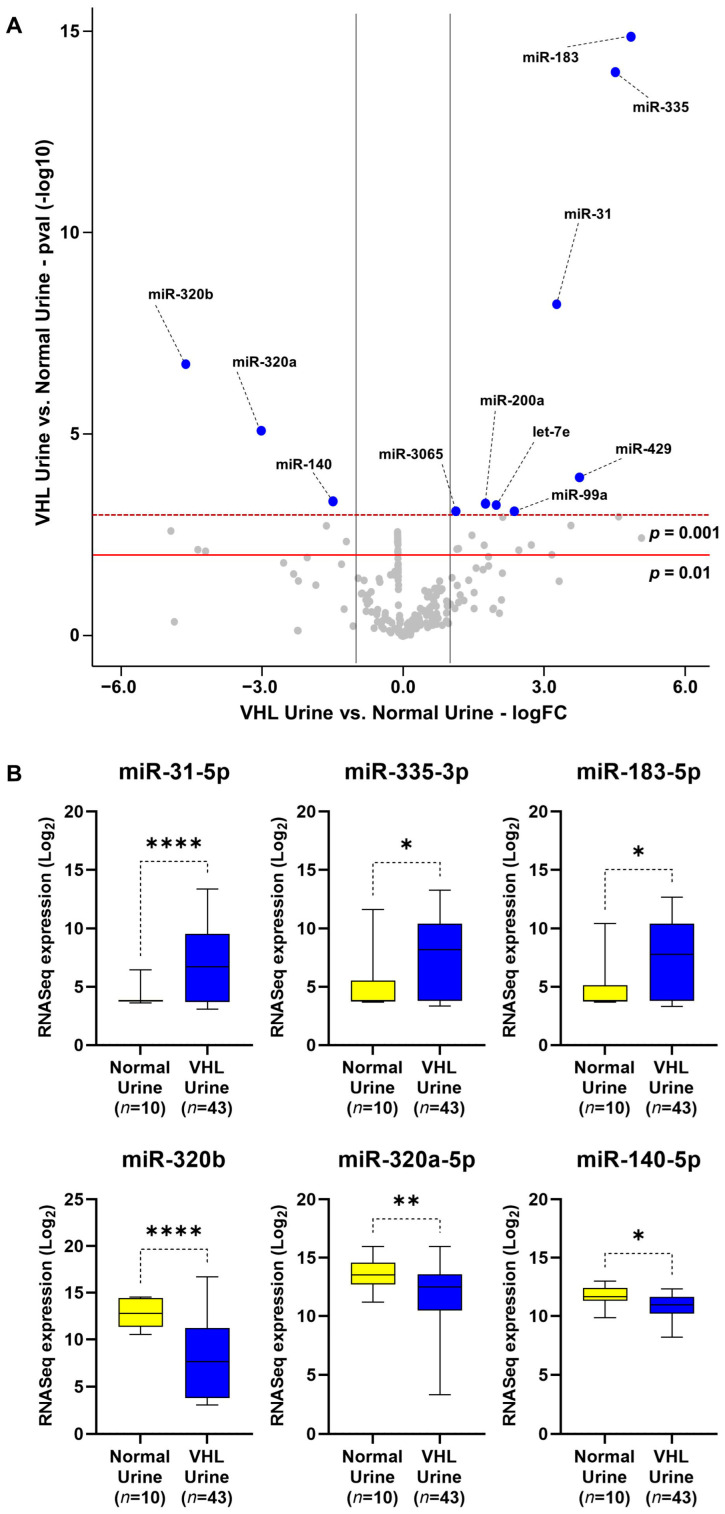
Analysis of differentially expressed miRNA between VHL patients and normal control urine samples. (**A**) Volcano plot analysis comparing the urinary exosome-derived miRNAs from 10 normal controls against 43 VHL patient samples. MiRNAs with *p*-values > 0.001 and fold changes > 2 are labeled in blue, all other miRNAs are labeled in gray. (**B**) The three most differentially altered up- and down-regulated miRNAs were confirmed using Welch’s *t*-test. The red line represents *p* = 0.01, and the dashed dark red line represents *p* = 0.001. The gray lines represent log_2_ fold-changes of −1.0 and 1.0. * *p*-value < 0.05, ** *p*-value < 0.01, **** *p*-value < 0.0001.

Initial comparative analysis of all VHL urine samples versus control urine samples identified eleven miRNAs with significant alterations (fold change > 2.0, *p*-value < 0.001), consisting of eight upregulated miRNAs, including miR-183-5p, miR-335-5p, and miR-31-5p, and three downregulated miRNAs from the same family, miR-320b, miR-320a-5p, and miR-140-5p (Figure 2A). The significance of these top six miRNA differences was confirmed using an alternative statistical analysis (Figure 2B). These miRNAs highlighted distinct differences between the VHL urine and control urine samples but did not demonstrate differentiation between preoperative and postoperative VHL urine samples (Supplementary Figure S2).

### 3.4. Analysis of VHL-Associated ccRCC Tumor-Specific Urinary Exosomal miRNA Markers

**Figure 3:**
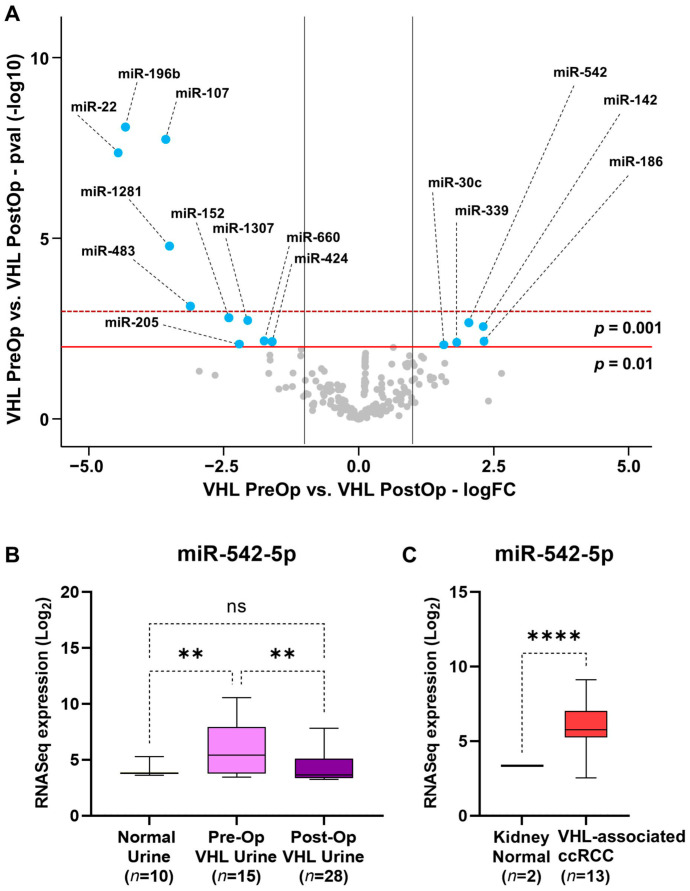
Analysis of differentially expressed miRNA between pre- and postoperative VHL urine samples. (**A**) Volcano plot analysis comparing the urinary exosome-derived miRNAs from 15 VHL preoperative samples against 28 postoperative samples. MiRNAs with *p*-values > 0.01 and fold changes > 2 are labeled in blue, all other miRNAs are labeled in gray. The red line represents *p* = 0.01, and the dashed dark red line represents *p* = 0.001. The gray lines represent log_2_ fold-changes of −1.0 and 1.0. (**B**,**C**) Analysis of miR-542-5p expression from urinary exosomes and from FFPE VHL-associated ccRCC and normal kidney, one-way ANOVA and Welch’s *t*-test, respectively. ** *p*-value < 0.01, **** *p*-value < 0.0001, ns—not significant.

To evaluate whether VHL-associated ccRCC tumor-specific exosomal miRNA could be detected, we initially compared the 15 preoperative urine samples to the 28 postoperative urine samples, assuming that the surgical excision of the ccRCC tumor would result in differential expression. With criteria of a fold change greater than 2 and a *p*-value greater than 0.01, 15 differentially expressed miRNAs were identified, consisting of 5 upregulated miRNAs and 10 downregulated miRNAs (Figure 3A). These data were compared to miRNA analysis of the ccRCC tumors derived from the same patients compared to normal kidney (Supplementary Figure S3). A single miRNA, miR-542-5p, was significantly upregulated (fold change > 2.0, *p*-value < 0.01) in both VHL-associated ccRCC tumors in comparison to normal kidneys and in preoperative VHL urine in comparison to postoperative urine (Figure 3A, Supplementary Figure S3 and Table S3). The statistical significance of this was confirmed, and it was shown that the preoperative VHL urine was significantly increased for miR-542-5p expression in comparison to both the normal control urine and the postoperative urine, while the normal control urine and the postoperative urine showed no significant difference (Figure 3B). The AUC comparing preoperative and postoperative urine samples was significant (*p* = 0.0031), but the AUC comparing control and preoperative urine samples did not reach significance (*p* = 0.067), suggesting analysis of a greater number of samples would be beneficial (Supplementary Figure S4).

Previous analyses of urinary miRNAs in sporadic ccRCC patients have identified several markers, including miR-210-5p and miR-15a-5p [17,24,25,26]. Additionally, increased miR-210-5p expression is a well-known consequence of VHL loss, and miR-15a-5p had upregulated expression in the excised tumors in this study (Supplementary Figure S3) [27]. Moreover, these two miRNAs were selected, and both demonstrated increased expression within VHL-associated ccRCC tumors, but no statistically significant increases in expression were observed in the VHL urinary exosomes, although a mild trend was observed with miR-210-5p (Supplementary Figures S3 and S5).

## 4. Discussion

Liquid biopsy is emerging as a powerful tool in oncology, including RCC, due to easy access to the necessary biofluids, such as urine and serum, via non-invasive or minimally invasive approaches [28,29,30,31]. This allows for rapid and cheap acquisition of the necessary testing materials that can be easily shipped and can practically overcome obstacles such as distance from a physical testing center [32,33]. The most important initial component is the identification of accurate, precise markers for a specific cancer type, such as VHL-associated ccRCC.

Management of VHL disease patients provides an exemplar for the usefulness of effective liquid biopsy technology due to the patient’s lifelong risk of developing multiple kidney tumors. Currently, VHL patients are imaged at reasonable intervals that involve a degree of expense and the ability of the patients to arrive at imaging facilities, which can leave patients with extensive gaps between assessments. The development of a non-invasive test utilizing readily available and transportable biofluids could provide a significant improvement in the management of VHL patients, improving early detection of kidney tumors and successful intervention. This could be integrated into the existing imaging regime if effective markers of VHL-associated ccRCC can be identified. Additionally, the loss of VHL as a common feature of sporadic ccRCC and markers identified in VHL-associated ccRCC may be applicable to the broader field of sporadic ccRCC, but this would require further comparative investigations.

This analysis highlighted a significant difference in the urinary exosome-derived miRNA expression profile between urine samples derived from VHL patients and those derived from normal control individuals. This included decreased expression of members of the miR-320 family, which are considered to have tumor suppressive activity [34]. Importantly, miR-320a has been shown to be suppressed by the HIF1α transcription factor, which is stabilized and uncontrollably activated as a result of VHL inactivation [35]. Notably, many of the differentially expressed miRNAs present in the VHL urinary exosomes did not differ between the pre- and postoperative urine samples, as might be expected if they represented a signal originating from the excised tissue. Due to the multifactorial nature of VHL, most of these patients would have other tumors, mostly benign, present in their bodies and likely have a greater number of kidney cysts than would be present in normal controls. These occurrences could also influence the urinary exosome content in these patients and contribute towards this distinct miRNA expression profile, particularly in the case of the HIF1α-repressed miR-320a. This would also correlate with the previous observations that miR-31 and miR-335 are downregulated in sporadic ccRCC, while they had increased expression in the VHL urine samples [36,37]. This highlights the scientific importance of being able to sample both pre- and postoperatively to allow for accurate designation of ccRCC-specific markers. The miR-542-5p miRNA demonstrated the perfect model for a potential marker of VHL-associated ccRCC as it was lowly expressed in normal control urinary exosomes, significantly increased in the preoperative urinary exosomes of tumor-bearing VHL patients, and subsequently reduced to normal levels of expression once the tumor had been removed. In concordance with this, the expression of miR-542-5p was increased in the VHL-associated ccRCC in comparison to the normal kidney. Increased expression of miR-542-5p has not been previously reported in sporadic or VHL-associated ccRCC, but increased expression has been observed as a driver of osteosarcoma. Further analysis is required before this could be considered a potential marker for VHL-associated ccRCC, but it shows the potential for useful markers is present.

These data also highlighted a high degree of variation between samples, even between multiple postoperative urine samples acquired from the same patient. This may represent actual variation within the patients or reflect inconsistencies with the methods for exosome isolation. The methodologies for isolating, purifying, and validating exosomes are advancing but yet to be fully refined, and this may result in more consistent results in the future. Additionally, this study was performed on a limited number of samples due to the relative rarity of VHL patients. This limitation can be addressed by further future analysis of larger cohorts of pre- and postoperatively acquired urine samples now that initial data demonstrate the potential importance of the analysis. Increased patient numbers would also aid in addressing some of the variation seen within samples. Other limitations in this study were the lack of validation of findings by a secondary methodology and a lack of direct comparison to sporadic ccRCC. Secondary validation by alternative methodology was confounded by the necessity to utilize the majority of collected materials for the initial study, and greater material collection would be necessary in future studies. Now that this initial study has demonstrated the potential for VHL-associated ccRCC miRNA biomarkers, comparison to sporadic ccRCC in further studies will allow for the ascertainment of any markers that are universal to ccRCC or specific VHL-associated ccRCC.

This study has shown the potential for miRNA profiling of exosomes from readily available biofluids to both distinguish VHL patient urine from normal control urine microRNAs and provide a biomarker for the presence of VHL-associated ccRCC. Further studies are necessary to validate these results within larger and more consistent cohorts and demonstrate the utility of urinary exosome-derived miRNAs as biomarkers in kidney cancer.

## Figures and Tables

**Table 1:** Clinicopathological features of the patients and samples analyzed.

Variable	*n*	(%)	Total
Age (years)			
Mean ± SD	45 ± 14 yr		
Range	21–72 yr		
Gender			
Male	20	58.8	34
Female	14	41.2	
Highest nuclear grade (WHO/ISUP)			
2	25	73.5	34
3	8	23.5	
4	1	3.7	
Metastasis			
Yes	3	8.8	34
No	31	91.2	
Years since first renal tumor treated		0.0	
less than 5	9	33.3	34
from 5 to 10 years	8	29.6	
from 11 to 20 years	15	55.6	
>20 years	2	7.4	
Other tumors manifestations			
Hemangioblastomas	14	41.0	34
Pancreas neuroendocrine tumor	2	6.0	
Endolymphatic sac tumor	2	6.0	
Epidermal cyst inclusion	1	3.0	
Parathyroid tumor	1	3.0	
Not specified in medical records	14	41.0	

## Data Availability

The original contributions presented in the study are included in the article/Supplementary Materials, further inquiries can be directed to the corresponding author.

## Supplementary Materials

The following supporting information can be downloaded at: https://www.mdpi.com/article/10.3390/genes15070905/s1, Figure S1: Characterization of Exosome Isolates.; Figure S2: Pre- and Postoperative VHL Urine analysis for marker miRNAs.; Figure S3: Differential miRNA expression in VHL-associated ccRCCs.; Figure S4: ROC curves for miR-542-5p; Figure S5: Pre- and postoperative VHL urine analysis for known marker miRNAs associated with ccRCC; Table S1: Data collected with the urine samples. Urine collection, processing, and storage.; Table S2: Collated results for Exo-Check® exosome array.; Table S3: Selected miRNAs from comparative analyses with FC > 2.0 and *p*-value < 0.01.
