# Supplementary material for: Evaluating the Urinary Exosome microRNA Profile of von Hippel Lindau Syndrome Patients with Clear Cell Renal Cell Carcinoma
Source: Genes (Basel). 2024 Jul 11;15(7):905. doi: 10.3390/genes15070905 (PMC11276299; doi:10.3390/genes15070905)
Supplement: Supplementary file 1 [file genes-15-00905-s001.zip › genes-3084732-supplementary.pdf]

**URINE**
ID
volume in mL
date of collection
date of processing x CFU
vials
**COLLECTION:**
pretreatment (Prostate massage/ catheter) NONE
Ethical Y/N
collection method (Mid-stream)/catheter
collection time
collection date
volume collected
collection device
**STORAGE**
after collection store at 4ºC
Transfer to the lab in ice Y/N
arrived at the lab date
hours passed after collection <4h/ <6h
light protection Y/N
Exclusion macrohematuria Y/N
quality -pH Y/N
quality protein level Y/N
**PROCESSING**
1) homogenize sample before processing Y/N
2) urine centrifugation 3000rpm x 15 min=704 G force
3) centrifugation at 4ºC Y/N
4) supernatant: loose pellet pipetting without disturbs Y/N
5) cell pellet stored Y/N
6) register # aliquots CFU
7) Storage at -80ºC/ location
8) method freeze: quick -80ºC
9) Defrost: in Room temp water bath until defrost

Supplementary Table S1: Data collected with the urine samples. Urine collection, processing, and storage.

Position	ID	Description	VHL Urine					Control Urine		
A1	Positive Ctl.	N/A	N/A	N/A	N/A	N/A	N/A	N/A	N/A	N/A
A2	GM130	Cis-golgi Matrix Protein	++	+	++	+	N/A	+	N/A	+
A3	FLOT1	Flotillin-1	+	+	+	-	-	+	-	+
A4	ICAM1	Intercellular Adhesion Molecule 1	+++	+++	++	++	++	+++	-	+++
A5	ALIX	Programmed Cell Death 6 Interacting Protein	++	+	+	+	+	++	-	+
A6	CD81	Tetraspanin	+	+	+	+	+	+	+	+
B1	CD63	Tetraspanin	+++	+++	+++	+++	+++	+++	+	+++
B2	EpCAM	Epithelial Cell Adhesion Molecule	+	-	+	-	-	+	-	-
B3	ANXA5	Annexin A5	+++	+++	+++	+++	++	+++	-	+++
B4	TSG101	Tumor Susceptibility Gene 101	+++	+++	++	+++	++	+++	-	+++
B5	Blank	N/A	N/A	N/A	N/A	N/A	N/A	N/A	N/A	N/A
B6	Positive Ctl.	N/A	N/A	N/A	N/A	N/A	N/A	N/A	N/A	N/A

Supplementary Table S2: Collated results for Exo-Check® exosome array.

CD81 and CD63 were expressed in all the samples.

miRNA	Comparison	Fold change	Fold change (log2)	p-value	Adjusted p-value
hsa-miR-25-5p	All VHL Urine vs. Normal Urine	33.579	5.069	0.00366812	0.011658735
hsa-miR-183-5p	All VHL Urine vs. Normal Urine	28.702	4.843	1.27945E-15	4.04307E-13
hsa-miR-182-5p	All VHL Urine vs. Normal Urine	23.972	4.583	0.00108442	0.010659708
hsa-miR-335-5p	All VHL Urine vs. Normal Urine	22.809	4.512	9.9528E-15	1.57254E-12
hsa-miR-429	All VHL Urine vs. Normal Urine	13.549	3.760	0.000113908	0.005999152
hsa-miR-241-5p	All VHL Urine vs. Normal Urine	11.866	3.569	0.001793835	0.010659708
hsa-miR-31-5p	All VHL Urine vs. Normal Urine	9.679	3.275	5.60748E-09	5.90655E-07
hsa-miR-1281-5p	All VHL Urine vs. Normal Urine	8.960	3.164	0.009559349	0.024558977
hsa-miR-148b-5p	All VHL Urine vs. Normal Urine	6.620	2.727	0.005461802	0.015273712
hsa-miR-361-5p	All VHL Urine vs. Normal Urine	5.505	2.461	0.007342814	0.019498564
hsa-miR-99a-5p	All VHL Urine vs. Normal Urine	5.164	2.368	0.000800252	0.010659708
has-let-7g-5p	All VHL Urine vs. Normal Urine	4.345	2.119	0.001113487	0.010659708
has-let-7e-5p	All VHL Urine vs. Normal Urine	3.953	1.983	0.000562593	0.010659708
hsa-miR-200a-5p	All VHL Urine vs. Normal Urine	3.374	1.755	0.000512254	0.010659708
hsa-miR-146a-5p	All VHL Urine vs. Normal Urine	3.305	1.725	0.00554204	0.015362145
hsa-miR-1269a	All VHL Urine vs. Normal Urine	2.760	1.465	0.003138054	0.011141855
has-let-7f-5p	All VHL Urine vs. Normal Urine	2.263	1.178	0.006737131	0.018512464
hsa-miR-144-5p	All VHL Urine vs. Normal Urine	2.211	1.144	0.0069259	0.018867106
hsa-miR-3065-5p	All VHL Urine vs. Normal Urine	2.181	1.125	0.000805411	0.010659708
hsa-miR-139-5p	All VHL Urine vs. Normal Urine	-2.308	-1.207	0.00448479	0.013244801
hsa-miR-140-5p	All VHL Urine vs. Normal Urine	-2.804	-1.488	0.000450757	0.010659708
hsa-miR-423-5p	All VHL Urine vs. Normal Urine	-3.097	-1.631	0.001827433	0.010659708
hsa-miR-320a-5p	All VHL Urine vs. Normal Urine	-8.055	-3.010	7.99371E-06	0.000505202
hsa-miR-486-5p	All VHL Urine vs. Normal Urine	-18.390	-4.201	0.007812706	0.020573458
hsa-miR-23a-5p	All VHL Urine vs. Normal Urine	-20.598	-4.364	0.007145916	0.019300081
hsa-miR-320b	All VHL Urine vs. Normal Urine	-24.592	-4.620	1.73401E-07	1.36987E-05
hsa-miR-320c	All VHL Urine vs. Normal Urine	-30.663	-4.938	0.002451932	0.010659708
hsa-miR-186-5p	PreOp VHL Urine vs. PostOp VHL Urine	4.995	2.320	0.006942929	0.068379087
hsa-miR-142-5p	PreOp VHL Urine vs. PostOp VHL Urine	4.965	2.312	0.002707284	0.068379087
hsa-miR-542-5p	PreOp VHL Urine vs. PostOp VHL Urine	4.116	2.041	0.002120531	0.068379087
hsa-miR-339-5p	PreOp VHL Urine vs. PostOp VHL Urine	3.528	1.819	0.007420709	0.068379087
hsa-miR-30c-5p	PreOp VHL Urine vs. PostOp VHL Urine	2.990	1.580	0.008631719	0.068379087
hsa-miR-424-5p	PreOp VHL Urine vs. PostOp VHL Urine	-3.034	-1.601	0.007158388	0.068379087
hsa-miR-660-5p	PreOp VHL Urine vs. PostOp VHL Urine	-3.353	-1.745	0.00681669	0.068379087
hsa-miR-1307-5p	PreOp VHL Urine vs. PostOp VHL Urine	-4.152	-2.054	0.001871645	0.068379087
hsa-miR-205-5p	PreOp VHL Urine vs. PostOp VHL Urine	-4.605	-2.203	0.008428092	0.068379087
hsa-miR-152-5p	PreOp VHL Urine vs. PostOp VHL Urine	-5.281	-2.401	0.001539034	0.068379087
hsa-miR-483-5p	PreOp VHL Urine vs. PostOp VHL Urine	-8.650	-3.113	0.00074045	0.04679644
hsa-miR-1281-5p	PreOp VHL Urine vs. PostOp VHL Urine	-11.290	-3.497	1.60436E-05	0.001267448
hsa-miR-107	PreOp VHL Urine vs. PostOp VHL Urine	-11.898	-3.573	1.79421E-08	2.83484E-06
hsa-miR-196b-5p	PreOp VHL Urine vs. PostOp VHL Urine	-19.890	-4.314	8.02039E-09	2.53444E-06
hsa-miR-22-5p	PreOp VHL Urine vs. PostOp VHL Urine	-21.897	-4.453	4.15856E-08	4.38035E-06
hsa-miR-148b-5p	VHL ccRCC tumor vs. Normal Kidney	214.497	7.745	1.36061E-06	0.000429952
hsa-miR-181a-5p	VHL ccRCC tumor vs. Normal Kidney	213.319	7.737	2.26995E-05	0.001024722
hsa-miR-19b1-5p	VHL ccRCC tumor vs. Normal Kidney	103.881	6.699	4.08949E-06	0.000448001
hsa-miR-19b2-5p	VHL ccRCC tumor vs. Normal Kidney	102.896	6.685	4.25318E-06	0.000448001
hsa-miR-15a-5p	VHL ccRCC tumor vs. Normal Kidney	97.931	6.614	7.75185E-06	0.000612396
hsa-miR-107	VHL ccRCC tumor vs. Normal Kidney	77.192	6.270	1.58782E-05	0.001003501
hsa-miR-15b-5p	VHL ccRCC tumor vs. Normal Kidney	68.018	6.088	2.23287E-05	0.001024722
hsa-miR-186-5p	VHL ccRCC tumor vs. Normal Kidney	57.945	5.857	3.42475E-05	0.001352774
hsa-miR-7-5p	VHL ccRCC tumor vs. Normal Kidney	53.111	5.731	0.000128474	0.004510866
hsa-miR-497-5p	VHL ccRCC tumor vs. Normal Kidney	43.863	5.455	0.0038488	0.079281287
hsa-miR-185-5p	VHL ccRCC tumor vs. Normal Kidney	36.374	5.185	0.001186854	0.037504587
hsa-miR-455-5p	VHL ccRCC tumor vs. Normal Kidney	25.307	4.661	0.006334346	0.111202963
hsa-miR-542-5p	VHL ccRCC tumor vs. Normal Kidney	18.784	4.231	0.003764689	0.079281287
hsa-miR-320b	VHL ccRCC tumor vs. Normal Kidney	14.563	3.864	0.003901437	0.079281287
hsa-miR-615-5p	VHL ccRCC tumor vs. Normal Kidney	14.117	3.819	0.003414275	0.079281287
hsa-miR-629-5p	VHL ccRCC tumor vs. Normal Kidney	10.610	3.407	0.003845643	0.079281287
hsa-miR-126-5p	VHL ccRCC tumor vs. Normal Kidney	10.395	3.378	0.009019775	0.137601969
hsa-miR-365a-5p	VHL ccRCC tumor vs. Normal Kidney	8.835	3.143	0.008617076	0.137601969
hsa-miR-338-5p	VHL ccRCC tumor vs. Normal Kidney	8.457	3.080	0.009381057	0.137601969
hsa-miR-891a-5p	VHL ccRCC tumor vs. Normal Kidney	-54.119	-5.758	0.004014242	0.079281287
hsa-miR-9-5p	VHL ccRCC tumor vs. Normal Kidney	-88.233	-6.463	0.005560667	0.103362988
hsa-miR-206	VHL ccRCC tumor vs. Normal Kidney	-122.508	-6.937	0.009579884	0.137601969

Supplementary Table S3: Selected miRNAs from comparative analyses with FC>2.0 and p-value <0.01.

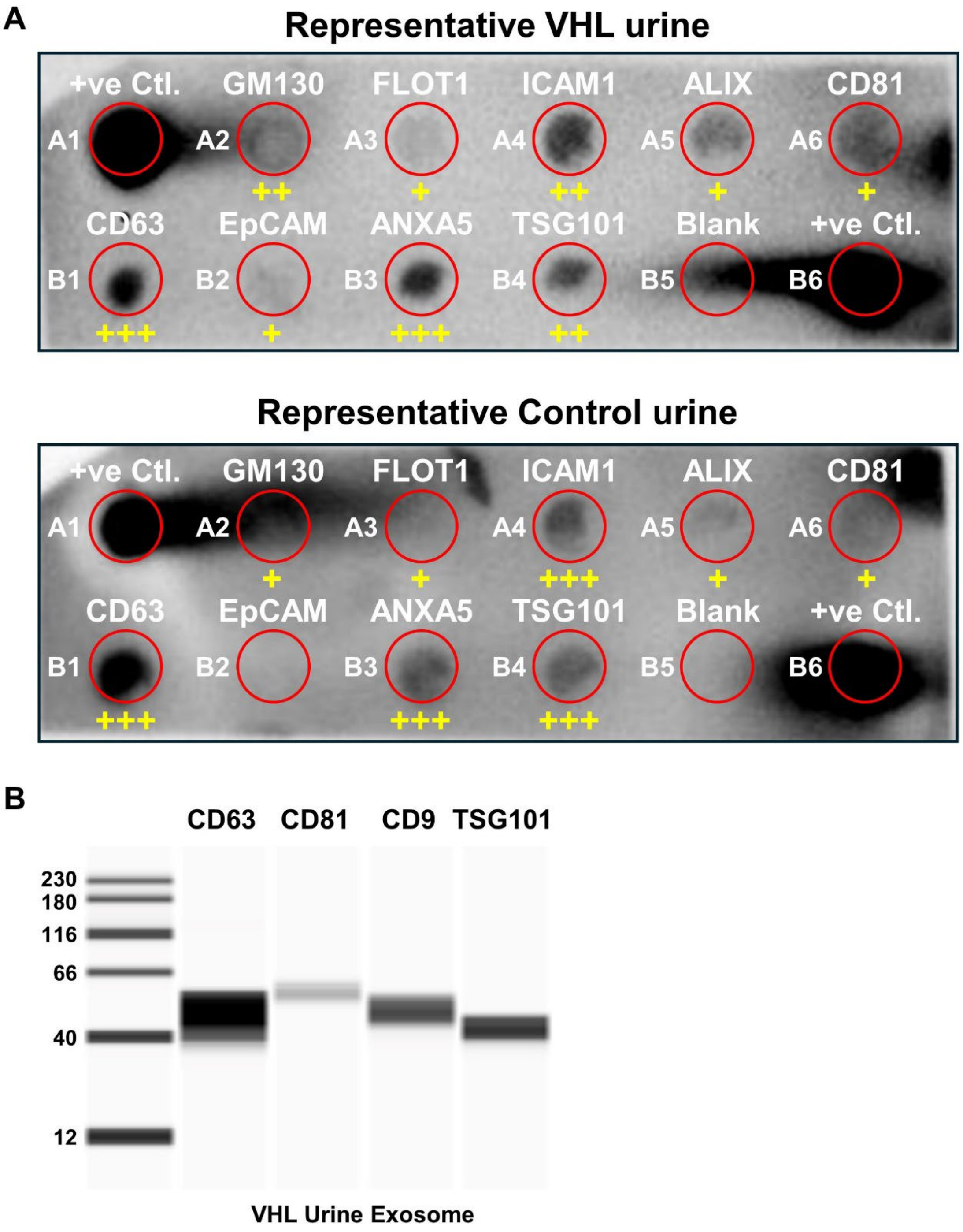

Supplementary Figure S1: Characterization of Exosome Isolates.

A) Representative VHL-urine and control urine samples for Exosome characterization by Exo-Check® exosome array.

B) Western blot analysis of CD63, CD81, CD9, and TSG101 antibodies for an example of a VHL urine exosome sample.

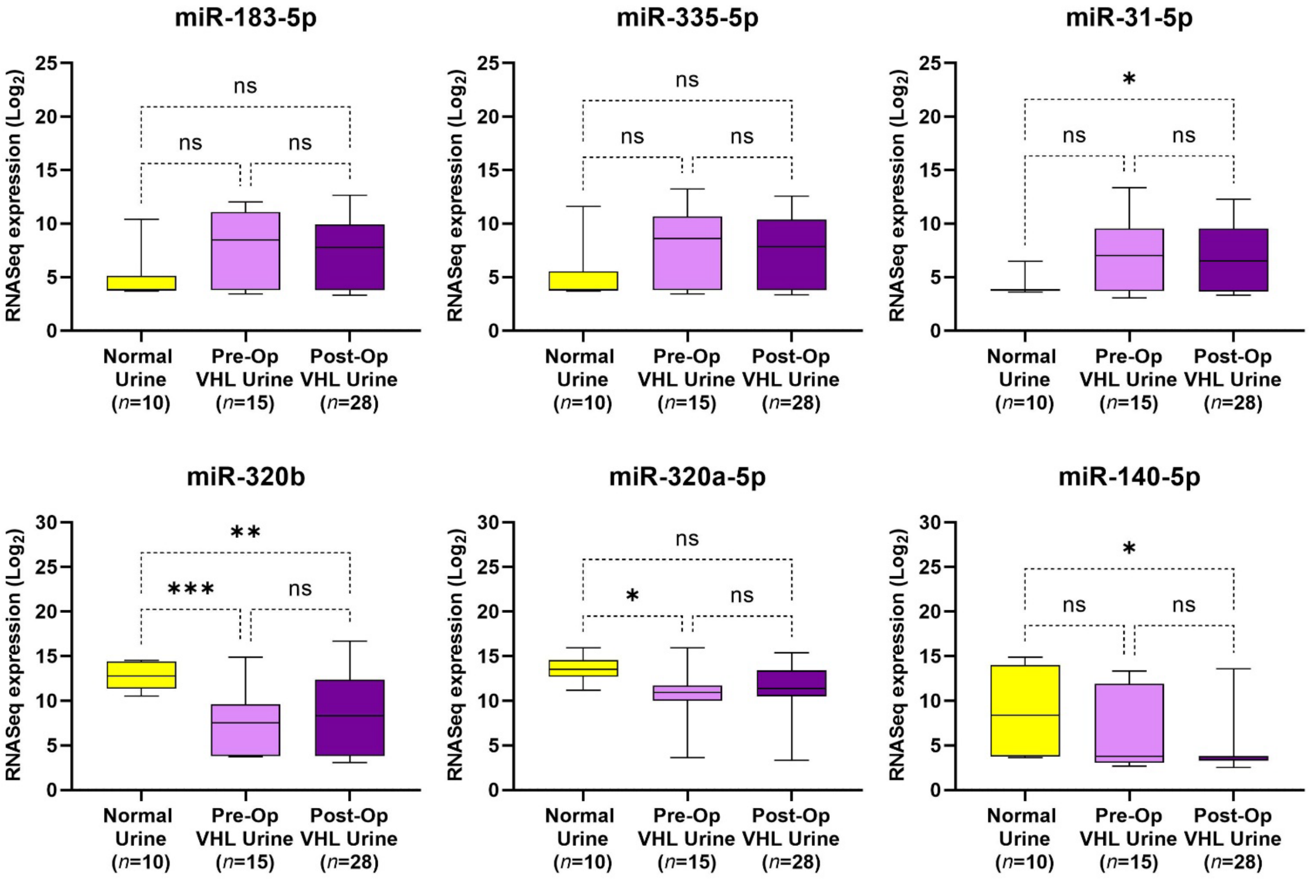

Supplementary Figure S2: Pre- and Postoperative VHL Urine analysis for marker miRNAs.

Confirmatory box plots for six selected up- and downregulated miRNAs comparing normal urine exosomes to VHL urine exosomes, with the VHL urines separated into pre- and postoperative urines, one-way ANOVA. * *p*-value <0.05, ** *p*-value <0.01, *** *p*-value <0.001, ns – not significant

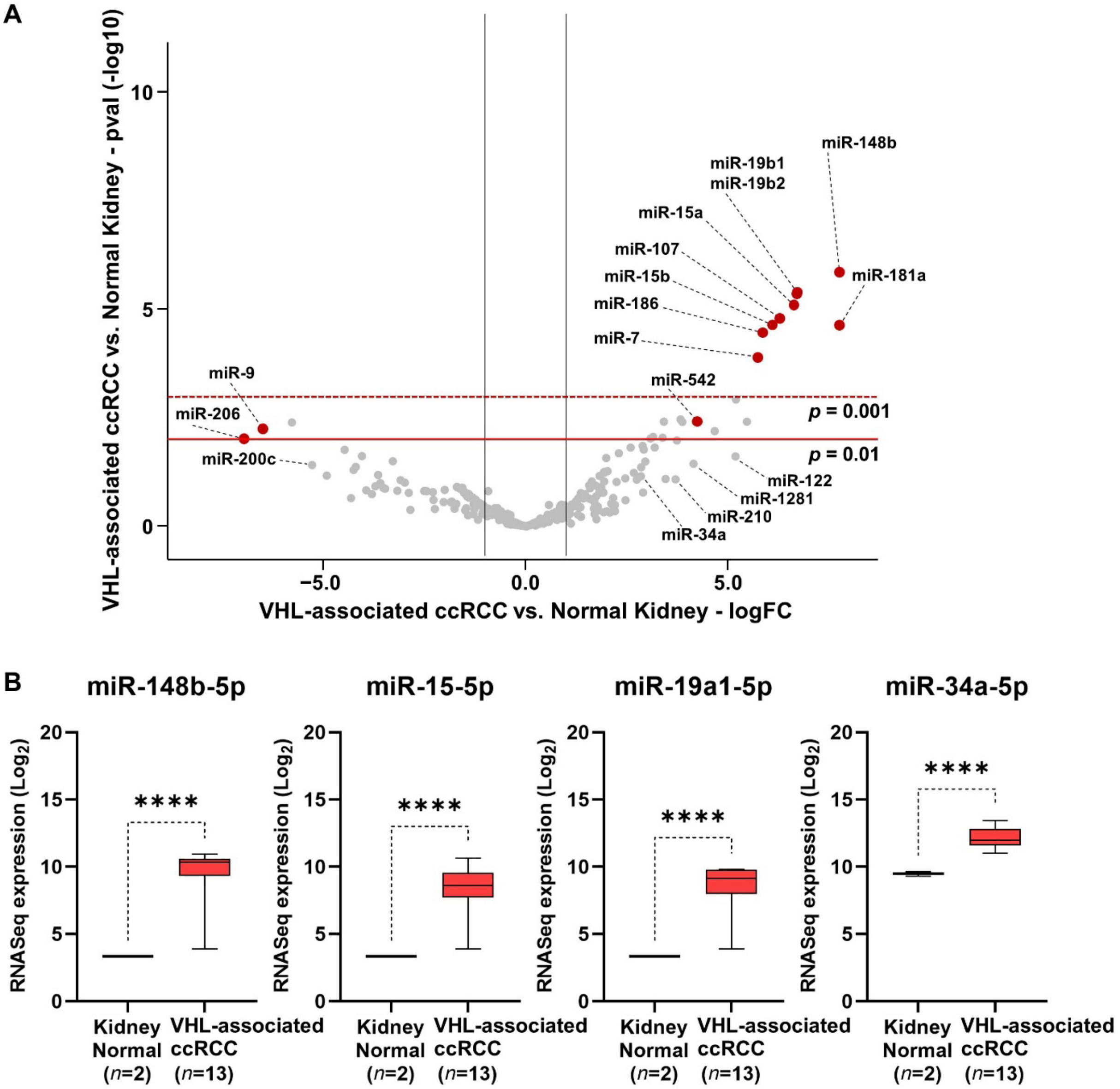

Supplementary Figure S3: Differential miRNA expression in VHL-associated ccRCCs

A) Volcano plot analysis comparing 13 VHL-associated ccRCC samples excised from 13 VHL patients within this study compared to normal kidney, consisting of an in-house pool of microdissected FFPE normal kidney. Red line represents p=0.01 and dashed dark red line represents p=0.001. MiRNAs with p-values > 0.001 and fold changes > 2 are labeled in red, all other miRNAs are labeled in gray. The gray lines represent log2 fold-changes of −1.0 and 1.0. B) Selected miRNAs, including miR-15a-5p, with significantly increased expression in VHL-associated ccRCC in comparison to normal kidney, Welch's t-test. **** p-value <0.0001

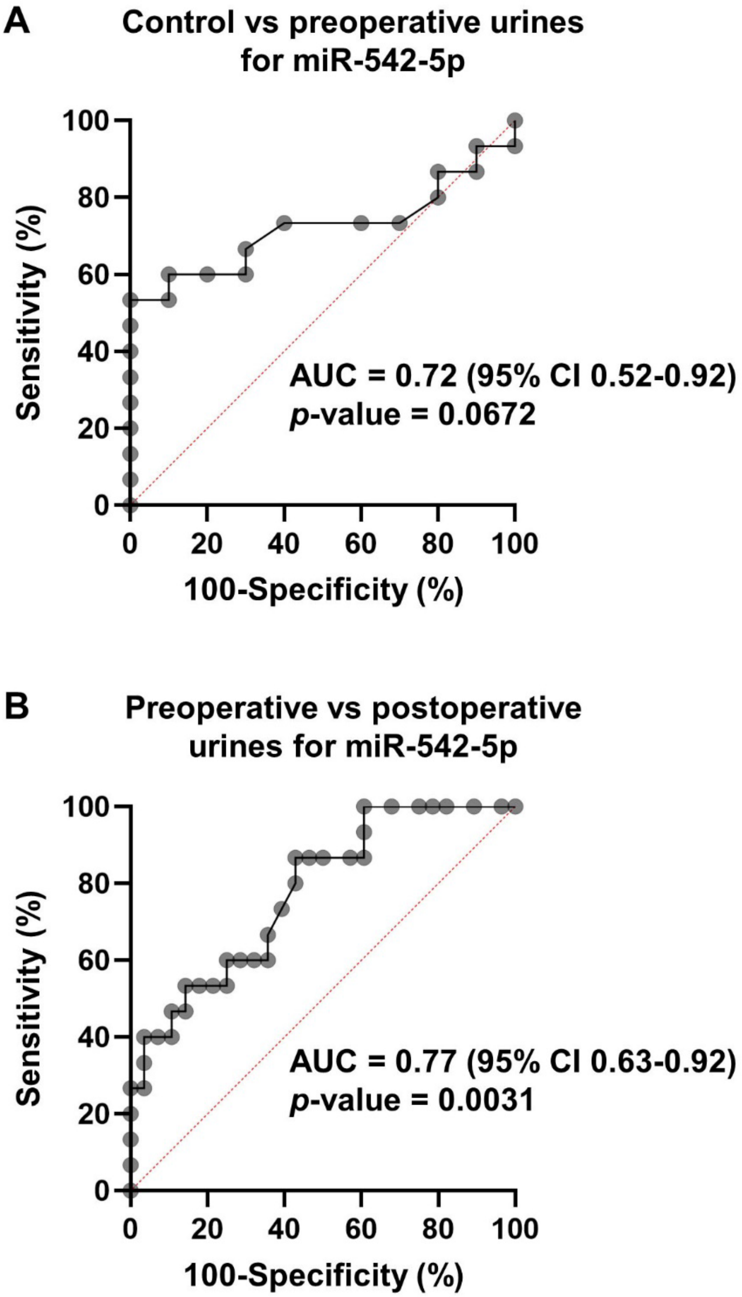

Supplementary Figure S4. ROC curves for miR-542-5p.

Receiver operating characteristic (ROC) curves for miR-542-5p demonstrating the comparison between preoperative urines and control urines (A) and between preoperative urines and postoperative urines (B).

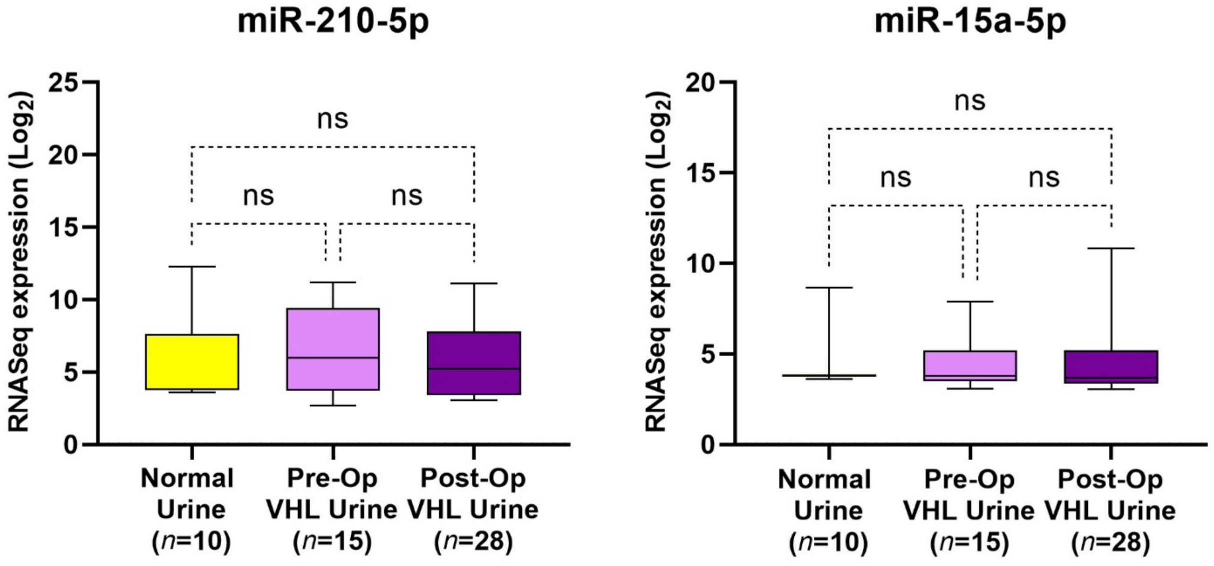

Supplementary Figure S5. Pre- and postoperative VHL urine analysis for known marker miRNAs associated with ccRCC.

Box plots comparing the expression of known miRNA markers of sporadic ccRCC within the normal urine exosomes and thew VHL urine exosomal miRNA, with the VHL urines separated into pre- and postoperative urines, one-way ANOVA. ns – not significant
